# Deletion of the Notch ligand Jagged1 during cochlear maturation leads to inner hair cell defects and hearing loss

**DOI:** 10.1038/s41419-022-05380-w

**Published:** 2022-11-18

**Authors:** Felicia A. Gilels, Jun Wang, Anwen Bullen, Patricia M. White, Amy E. Kiernan

**Affiliations:** 1 Department of Pathology and Laboratory Medicine, University of Rochester Medical Center, Rochester, NY 14642 USA; 2 Department of Ophthalmology, University of Rochester Medical Center, Rochester, NY 14642 USA; 3 UCL Ear Institute, Faculty of Brain Sciences, University College London, 332 Gray’s Inn Road, London, WC1X 8EE UK; 4 Department of Neuroscience, Ernest J. Del Monte Institute for Neuroscience, University of Rochester Medical Center, Rochester, NY 14642 USA; 5 Department of Biomedical Genetics, University of Rochester Medical Center, Rochester, NY 14642 USA

**Keywords:** Disease model, Cochlea

## Abstract

The mammalian cochlea is an exceptionally well-organized epithelium composed of hair cells, supporting cells, and innervating neurons. Loss or defects in any of these cell types, particularly the specialized sensory hair cells, leads to deafness. The Notch pathway is known to play a critical role in the decision to become either a hair cell or a supporting cell during embryogenesis; however, little is known about how Notch functions later during cochlear maturation. Uniquely amongst Notch ligands, Jagged1 (JAG1) is localized to supporting cells during cell fate acquisition and continues to be expressed into adulthood. Here, we demonstrate that JAG1 in maturing cochlear supporting cells is essential for normal cochlear function. Specifically, we show that deletion of JAG1 during cochlear maturation disrupts the inner hair cell pathway and leads to a type of deafness clinically similar to auditory neuropathy. Common pathologies associated with disruptions in inner hair cell function, including loss of hair cells, synapses, or auditory neurons, were not observed in JAG1 mutant cochleae. Instead, RNA-seq analysis of JAG1-deficient cochleae identified dysregulation of the Rho GTPase pathway, known to be involved in stereocilia development and maintenance. Interestingly, the overexpression of one of the altered genes, *Diaph3*, is responsible for autosomal dominant auditory neuropathy-1 (AUNA1) in humans and mice, and is associated with defects in the inner hair cell stereocilia. Strikingly, ultrastructural analyses of JAG1-deleted cochleae revealed stereocilia defects in inner hair cells, including fused and elongated bundles, that were similar to those stereocilia defects reported in AUNA1 mice. Taken together, these data indicate a novel role for Notch signaling in normal hearing development through maintaining stereocilia integrity of the inner hair cells during cochlear maturation.

## Introduction

The mammalian cochlear epithelia (the organ of Corti) is composed of sensory hair cells, supporting cells, and innervating neurons. The hair cells are mechanosensitive cells that display organized, hair-like structures atop their surface called stereocilia. There are two types of hair cells: inner hair cells, which are the primary sensory receptors, and outer hair cells, which amplify the auditory signal. In mammals these cells cannot regenerate; thus, damage to these cell types leads to deafness. Notch signaling plays multiple essential roles in the embryonic development of the inner ear sensory regions [1, 2]. This evolutionarily conserved signaling pathway functions through the interactions of membrane-bound ligands (Jagged-1–2, and Delta-like1,3-4) and receptors (NOTCH1-4). These cell-cell interactions trigger the cleavage and subsequent release of the activated form of Notch (the Notch Intracellular Domain or NICD), which translocates to the nucleus and interacts with the effector RBPJ (RBPjκ or CSL (CBF1, Suppressor of Hairless, Lag-1)) and alters transcription [3, 4].

During embryonic inner ear sensory development, Notch signaling functions reiteratively through two distinct signaling modalities: lateral induction and lateral inhibition [2, 5] (1). Initially, through the process of lateral induction [6], Notch signaling, via the JAG1 ligand, establishes the prosensory progenitors that give rise to both hair cells and supporting cells [7–10]. Subsequently, Notch functions during lateral inhibition via the DLL1 and JAG2 ligands, a process that creates the mosaic patterning of hair cells and supporting cells in the cochlea [11–13]. Despite these important early roles for Notch in inner ear development, there is a limited understanding of Notch function after birth during the cochlear maturation period. The continued expression pattern of one of the Notch ligands, JAG1, after birth and into adulthood, makes it a compelling candidate to function in the maturing cochlea [11, 14–16].

To investigate the role of the Notch ligand, JAG1, in the maturing cochlea, we conditionally deleted JAG1 in the early postnatal cochlea. We found that deletion of *Jag1* in neonatal supporting cells (*Sox2*^*CreER/+*^*Jag1*^*fl/fl*^) resulted in a specific form of hearing loss at 6 weeks that is clinically similar to auditory neuropathy. Histological analyses failed to reveal common defects for the type of hearing loss observed in JAG1-deficient mice, including loss of inner hair cell synapses or auditory neurons. RNA-seq analysis between JAG1-deleted and *Sox2*^*+/+*^ littermate control cochleae at postnatal day (P)6 indicated defects in the Rho GTPase signaling pathway, which is involved in actin regulation. Consistent with this, ultrastructural analyses revealed defects specifically in the inner hair cell stereocilia. Taken together, our results demonstrate that JAG1 signaling in maturing cochlear supporting cells is essential for normal cochlear function and indicates a novel role for JAG1 in supporting cell/hair cell interactions and stereocilia integrity.

## Materials and methods

### Animals and tamoxifen treatment

All experimental procedures were performed in accordance with guidelines and regulations of the University of Rochester Medical Center and the Guide for the Care and Use of Laboratory Animals of the National Institutes of Health. All animal experiments were approved by the University of Rochester’s Committee on Animal Resources. Mouse strains: *Sox2-Cre*^*ERT2*^; C57bl6/J background [17], *Jag1*^*flox*^; FVB/n background [18]. PCR primers: *Sox2-Cre*^*ERT2*^: CreF (5’ TGA TGA GGT TCG CAA GAA CC) and CreR (5’ CCA TGA GTG AAC GAA CCT GG) yielding a 350 bp band. *Jag1*^*flox*^: Jag1F (5’ AGG TTG GCC ACC TCT AAA TC) and Jag1R (5’ GCA AGT CTG TCT GCT TTC ATC), yielding a 316 bp band. The day of birth was considered postnatal day (P)0. All pups were given a single intraperitoneal injection of tamoxifen (Sigma, St. Louis, MO, USA; 75 µg/g body weight) on (P)0 and P1. Both *Sox2*^*+/+*^*Jag1*^*+/fl*^ and *Sox2*^*+/+*^*Jag1*^*fl/fl*^ mice were used, and are collectively referred to as *Sox2*^*+/+*^ mice. *Sox2*^*+/+*^ and *Sox2*^*CreER/+*^*Jag1*^*+/fl*^ littermates are used as controls throughout this study. Animals were collected at either P6 or 6 weeks of age. Mice received food and water ad libitum and were housed on a 12-h light to dark cycle. The number of biological replicates is reported in the figure legend for each analysis and were taken from at least two different litters. Roughly equal numbers of both sexes of mice were used in this study. Animals were randomly assigned to each analysis and no animals were excluded in this study.

### Auditory testing

6-week-old mice were anesthetized with an intraperitoneal injection of ketamine (80 mg/kg) in a sterile acepromazine/saline mixture (3 mg/kg). Auditory testing was conducted using a Smart EP Universal Smart Box (Intelligent Hearing Systems, Miami, FL, USA) as previously described [19].

### Tissue preparation and immunostaining

Cochleae were fixed overnight at 4 °C in 4% paraformaldehyde (PFA) (Santa Cruz Biotechnology, Dallas, TX, sc-281692). Adult tissue was decalcified in 0.2 M EDTA (pH 7.3) for 14 days at 4 °C. For whole-mount antigen retrieval, dissected cochleae were immersed in 30% sucrose, flash frozen in liquid nitrogen, allowed to thaw, washed in PBS, and blocked for one hour in 1% Triton X-100/5% horse serum (Sigma) in PBS, and incubated in primary antibodies overnight. Refer to Table S1 for information regarding primary antibodies used in this study. Tissue was subsequently washed in PBS and incubated for two hours at room temperature in Alexa Fluor 647 Phalloidin (Invitrogen, A22287), [1:100]) Alexa Fluor secondary antibodies (Invitrogen, ab150073, ab 11056, ab21202, and ab150074, [1:1000]), counterstained with 4‘,6-diamidino-2-phenylindole (DAPI), washed again in PBS and mounted in Fluorogel in Tris buffer (Electron Microscopy Sciences).

### Paraffin sections

Tissue was dehydrated through a series of EtOH washes from 70% to 100%, cleared in xylene, embedded in paraffin, and sectioned at a thickness of 7 µm. Slides were deparaffinized through a series of xylene and ethanol washes. Antigen retrieval was performed prior to immunostaining by incubating in 10 mM Sodium Citrate Buffer (pH 6) for 20 minutes at 98 °C and incubated overnight in primary and secondary antibodies at 4 °C.

### Frozen sections

Tissue was cryoprotected overnight at 4 °C in increasing concentrations of sucrose in PBS up to 30%, embedded in tissue freezing medium, frozen on dry ice, and sectioned at a thickness of 16 µm.

### Plastic sections

Cochlea were fixed in 0.1 M sodium cacodylate-buffered 2.5% glutaraldehyde/4% PFA overnight, decalcified for 1 week in 200 mM EDTA (PH 7.4), dehydrated with a series of ethanol washes, embedded in Technovit 7100 hardener (Kulzer), sectioned at 2.5 μm (HM 355 S Automatic Microtome), and stained with hematoxylin and eosin.

### Imaging

All sections were imaged on a Zeiss Axio microscope using Axiovision SE64 software. An Olympus FV1000 laser scanning confocal microscope (URMC Center for Advanced Light Microscopy and Nanoscopy (CALM) core) and a Nikon A1-R confocal microscope with Nikon NIS Elements software were used to capture Z-stack images of labeled cochleae in whole mount.

### Quantification

Quantification of hair cells, synapses, and supporting cells were performed on two confocal z-stack images taken from each cochlear region (apex, middle, base); and cells were manually quantified in two or three 300 µm segments per region in Affinity Photo (Serif Europe Ltd.). Spiral ganglion neurons were quantified in sections; a minimum of 30 sections were counted per animal and the area of Rosenthal’s canal was measured in the Axiovision SE64 software. The researcher was blinded as to the genotype for the quantification of all cell types and scoring of ABR/DPOAE thresholds.

### Statistical analysis

Before experiments were conducted, power calculations were performed to determine appropriate sample size and at least three individual mice per genotype were used per analysis. Statistics were performed in Prism9 (GraphPad) using standard functions. To ensure appropriate statistical tests were utilized, data from experiments designed to test differences between two groups (e.g., SGN’s per unit area (Fig. 3C)) were subjected to an F test to compare variance and a Shapiro-Wilk to test normality and were analyzed using unpaired two-tailed Welch’s *t*-test. To ensure appropriate statistical tests were utilized, data from experiments designed to detect differences among multiple groups and across multiple conditions (e.g., Auditory thresholds at multiple frequencies (Fig. 2A, B), ABR wave I absolute latencies at multiple sound pressure levels (Fig. 3D), IHC and OHC #’s at 6 weeks (Fig. 4I), quantification of synaptic components at multiple frequencies (Fig. 4J–L), ABR wave I amplitudes at increasing sound pressure levels (Fig. S2B), and quantification of hair cell and supporting cell subtypes at P6 (Fig. S3I)) were analyzed using a two-way ANOVA followed by Bonferroni’s post hoc tests. For these statistical tests, every possible comparison was made when relevant, and multiplicity adjusted *P* values are reported. In all cases, data met the assumptions of the statistical test used. *P* values <0.05 were considered statistically significant. Center values for each graph are defined as the mean and error bars are defined in each figure legend.

### RNA-seq

All pups were given a single intraperitoneal injection of tamoxifen (Sigma, St. Louis, MO, USA; 75 µg/g body weight) on (P)0 and P1. Cochlear tissues from P6 *Sox2*^*CreER/+*^*Jag1*^*fl/fl*^ and *Sox2*^*+/+*^ littermate controls (*n* = 6 each) were dissected in ice-cold DEPC-treated PBS and stored in RNAlater (Invitrogen, Carlsbad, CA, USA) at 4 °C. RNAs were purified by RNeasy Micro Kit (QIAGEN, Hilden, Germany). RNA quality and quantity was measured by a 2100 bioanalyzer (Agilent, Santa Clara, CA, USA). All RNAs had an RNA Integrity Number (RIN) value of 9.7–10. Illumina-compatible sequencing libraries were generated by the University of Rochester Genomics Research Center. Libraries were hybridized to the Illumina flow cell and single-end reads of 100nts were generated. Identification of significantly differentially expressed genes was determined using DeSeq2 (Bioconductor). The analysis package Ingenuity Pathway Analysis (IPA; QIAGEN) was used to determine whether particular pathways were significantly affected by the loss of JAG1.

### Scanning electron microscopy

Cochleae were fixed in 2.5% glutaraldehyde (Electron Microscopy Sciences, Hatfield, PA, USA) in 0.1 M phosphate buffer overnight at 4 °C with gentle rotation and processed using the OTOTO protocol [20]. Samples were dehydrated through a graded series of ethanol and critical point dried before mounting. Imaging was carried out on a JEOL 6700 F scanning electron microscope, operating at 5 kV in secondary electron detection mode.

## Results

### Deletion of the Notch ligand JAG1 in maturing supporting cells

**Fig. 1:**
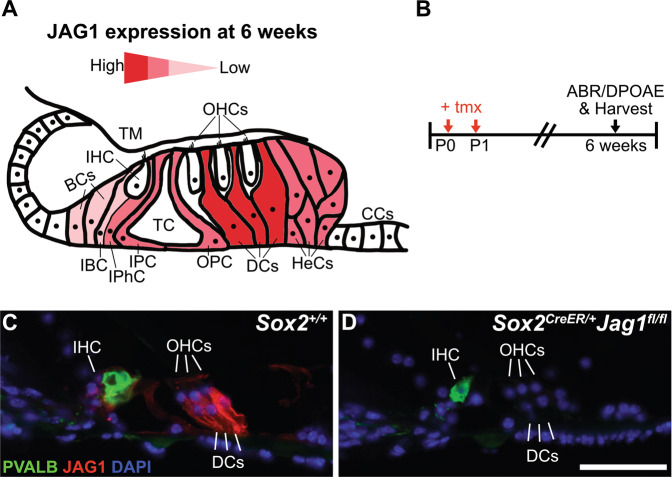
Deletion of the Notch ligand JAG1 in maturing supporting cells results in undetectable JAG1 immunostaining at 6 weeks. **A** Drawing of a cross-section through the adult organ of Corti showing JAG1 expression in supporting cells at 6 weeks of age. **B** Offspring from *Sox2*^*CreER/+*^*Jag*^*+/fl*^ x *Jag1*^*fl/fl*^ matings were administered tamoxifen (+tmx) at postnatal days (P)0 and P1; hearing was analyzed and cochleae were harvested at 6 weeks. **C**, **D** Paraffin sections through 6-week-old cochlea stained for hair cells (PVALB), nuclei (DAPI) and JAG1. Scale bar: 50 µm. **C** *Sox2*^*+/+*^ littermate control showing the normal expression of JAG1 in Deiters’ cells, inner phalangeal cells, and pillar cells, which is largely undetectable in *Sox2*^*CreER/+*^*Jag1*^*fl/fl*^ mutant cochleae (**D**).

To understand the role of JAG1 in the maturing cochlea, JAG1 was conditionally deleted in neonatal cochlear supporting cells using the tamoxifen-inducible *Sox2*^*CreER*^ mouse line [17] crossed to a *Jag1*^*flox*^ allele [18]. In the mouse cochlea, *Sox2*^*CreER*^ is expressed in all supporting cell subtypes [21], while JAG1 is localized to inner phalangeal cells, pillar cells and Deiters’ cells [16, 22] (Fig. 1A, C, red). Offspring from *Sox2*^*CreER*^*Jag1*^*+/fl*^ and *Jag1*^*fl/fl*^ crosses were injected with tamoxifen at postnatal day (P)0 and P1 (Fig. 1B) [23], a time when hair cells and supporting cells are maturing. *Sox2*^*+/+*^ mice referred to in this study are littermate controls. To determine the efficiency of deletion, immunohistochemistry was performed at 6 weeks to detect JAG1 protein. Results showed that JAG1 was largely undetectable by immunohistochemistry at 6 weeks (Fig. 1D, red), indicating that deletion was efficient. To determine how fast the deletion and protein reduction occurred, we performed immunohistochemistry at P6. In controls at P6, JAG1 is localized to supporting cells, including the greater epithelial ridge (GER), pillar cells and Deiters’ cells (Fig. S1A, C, red). In *Sox2*^*CreER/+*^*Jag1*^*fl/fl*^ cochleae at P6, significant downregulation of JAG1 protein was observed in all supporting cells (Fig. S1D, red), indicating that deletion and protein decline occurred rapidly. Together, these results indicate that JAG1 is efficiently deleted throughout the cochlea at early postnatal time points.

### Deletion of JAG1 in the maturing cochlea causes hearing loss

**Fig. 2:**
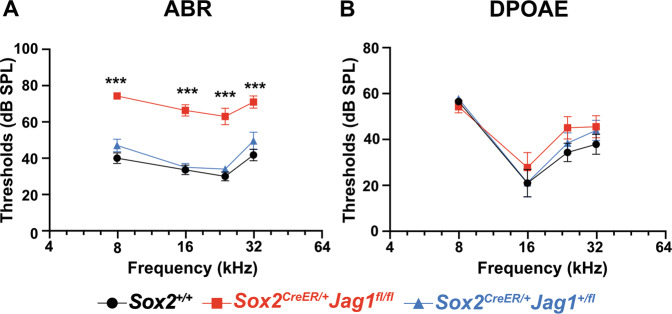
Deletion of *Jag1* in the maturing cochlea causes hearing loss. **A**, **B** Hearing test results at 6 weeks of age. Data expressed as mean ± SEM. **A** Average auditory brainstem response (ABR) thresholds for pure tone stimuli at 8, 16, 24, and 32 kHz showed significant increases in *Sox2*^*CreER/+*^*Jag1*^*fl/fl*^ mice. Significance (*) indicated *Sox2*^*+/+*^ vs. *Sox2*^*CreER/+*^*Jag1*^*fl/fl*^; two-way ANOVA Bonferroni adjusted; ***P ≤ 0.001. *Sox2*^*+/+*^ *n* = 11, *Sox2*^*CreER/+*^*Jag1*^*fl/fl*^ *n* = 12, *Sox2*^*CreER/+*^*Jag1*^*+/fl*^ *n* = 10. **B** Average distortion product otoacoustic emission (DPOAE) measurements showed no significant threshold differences between *Sox2*^*CreER/+*^*Jag1*^*fl/fl*^ and controls at all frequencies. Two-way ANOVA Bonferroni adjusted; *n* = 10 per genotype.

To determine the effects of JAG1 deletion on auditory function, we measured auditory brainstem responses (ABRs) and distortion product otoacoustic emissions (DPOAEs) in *Sox2*^*CreER/+*^*Jag1*^*fl/fl*^ mice and their littermate controls at 6 weeks of age (Fig. 2). Conditional deletion of *Jag1* in maturing supporting cells (*Sox2*^*CreER/+*^*Jag1*^*fl/fl*^) resulted in significantly elevated ABR thresholds across the 8–32 kHz frequency range (Fig. 2A, red). The threshold increases ranged from 30 to 50 dB, indicating compromised auditory function, but not complete deafness (*Sox2*^*CreER/+*^*Jag1*^*fl/fl*^; Fig. 2A, red). Littermates, in which only one copy of *Jag1* had been deleted (*Sox2*^*CreER/+*^*Jag1*^*+/fl*^; Fig. 2A, blue) were not significantly different from *Sox2*^*+/+*^ littermate controls (*Sox2*^*+/+*^; Fig. 2A, black), indicating that neither Cre expression, tamoxifen treatment, nor *Jag1* heterozygosity impacted ABR thresholds. In contrast to the ABRs, DPOAE thresholds were not significantly different between *Sox2*^*CreER/+*^*Jag1*^*fl/fl*^ mice and controls, indicating that cochlear amplification is preserved in JAG1-deficient mice and outer hair cell function is intact (Fig. 2B). Taken together, these results demonstrate that neonatal loss of JAG1 primarily affects the function of the inner hair cell pathway, resulting in a specific type of hearing loss that is clinically similar to auditory neuropathy [24]. To further characterize the hearing loss that we observed in *Sox2*^*CreER/+*^*Jag1*^*fl/fl*^ mice, we analyzed the ABR waveforms at 16 kHz in response to 75 dB SPL pure tone stimulus (Fig. S2A) and analyzed wave I amplitudes in response to pure tone stimuli of increasing sound pressure levels (Fig. S2B). The average ABR waveforms of JAG1-deficient mice (Fig. S2A, red) were considerably different from controls (Fig. S2A, black). In particular, *Sox2*^*CreER/+*^*Jag1*^*fl/fl*^ mice display a substantially decreased wave I response, which is generated by the signaling of inner hair cells to the auditory nerve [25]. Moreover, *Sox2*^*CreER/+*^*Jag1*^*fl/fl*^ cochleae do not display normal linear increases in wave I amplitudes in response increasing sound pressure levels (Fig. S2B, red), further indicating dysfunctional inner hair cell signaling. These results indicated that JAG1-deficient cochleae had defects in the inner hair cell pathway, which could include synaptopathies or neuropathies.

### JAG1-deficient mice demonstrate normal spiral ganglion neuron quantities and ABR wave I absolute latencies

**Fig. 3:**
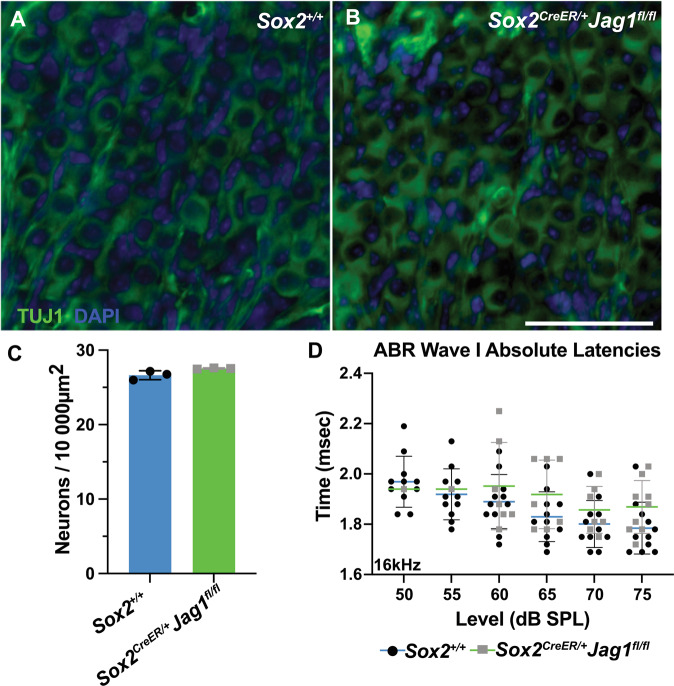
*Sox2*^*CreER/+*^*Jag1*^*fl/fl*^ mice have spiral ganglion neurons and normal ABR wave I absolute latencies. **A**, **B** Representative images of spiral ganglion neurons from single optical sections stained for anti-Tubulin β-III (TUJ1, green) and nuclei (DAPI, blue) in P6 *Sox2*^*+/+*^ littermate control (**A**) and *Sox2*^*CreER/+*^*Jag1*^*fl/fl*^ mutant mice (**B**). Scale bar: 50 µm. **C** Average number of spiral ganglion neurons (SGN) per unit area (10,000 µm^2^) in Rosenthal’s canal for P6 *Sox2*^*+/+*^ littermate control (black circles, blue mean bar) and *Sox2*^*CreER/+*^*Jag1*^*fl/fl*^ mutant (gray squares, green mean bar) mice are not significantly different. Data expressed as mean ± SD. Unpaired two-tailed Welch’s *t* test. *n* = 3 per genotype. **D** ABR wave I absolute latencies at 16 kHz for control (black circles, blue mean bar) mice and *Sox2*^*CreER/+*^*Jag1*^*fl/fl*^ mutants (gray squares, green mean bar) are not significantly different at 6 weeks. Data expressed as mean ± SD. Two-way ANOVA Bonferroni adjusted. *Sox2*^*+/+*^ *n* = 11, *Sox2*^*CreER/+*^*Jag1*^*fl/fl*^ *n* = 12.

Clinically, auditory neuropathies are a class of sensorineural hearing loss that are caused by pathology of the auditory nerve [24]. To determine whether spiral ganglion neuron loss contributed to the increased auditory thresholds observed in JAG1-deficient mice, we stained P6 *Sox2*^*CreER/+*^*Jag1*^*fl/fl*^ and *Sox2*^*+/+*^ littermate control cochlea for spiral ganglion neurons (Fig. 3A, B; TUJ1, green). Quantification did not reveal any significant differences between *Sox2*^*+/+*^ control (Fig. 3C, black circles) and *Sox2*^*CreER/+*^*Jag1*^*fl/fl*^ mice (Fig. 3C, gray squares), indicating that neuronal loss is unlikely to be the cause of increased auditory thresholds. However, auditory neuropathy can also be caused by demyelination diseases [26, 27] which cause slower nerve impulse conduction and demyelination of the auditory nerve would alter the peak latencies of the ABR waveform [28, 29]. To determine the effect of JAG1 deletion on auditory nerve conductance, we plotted absolute latencies of wave I of the 16 kHz ABR waveform at 6 weeks [30]. ABR wave I absolute latencies in *Sox2*^*CreER/+*^*Jag1*^*fl/fl*^ mice (Fig. 3D, gray squares) was not significantly different from *Sox2*^*+/+*^ littermate controls (Fig. 3D, black circles) at 6 weeks of age, suggesting that neonatal JAG1 deletion does not alter auditory nerve myelination [28, 29].

### Inner hair cells are present and their synapses are largely unaffected in JAG1-deleted cochleae

**Fig. 4:**
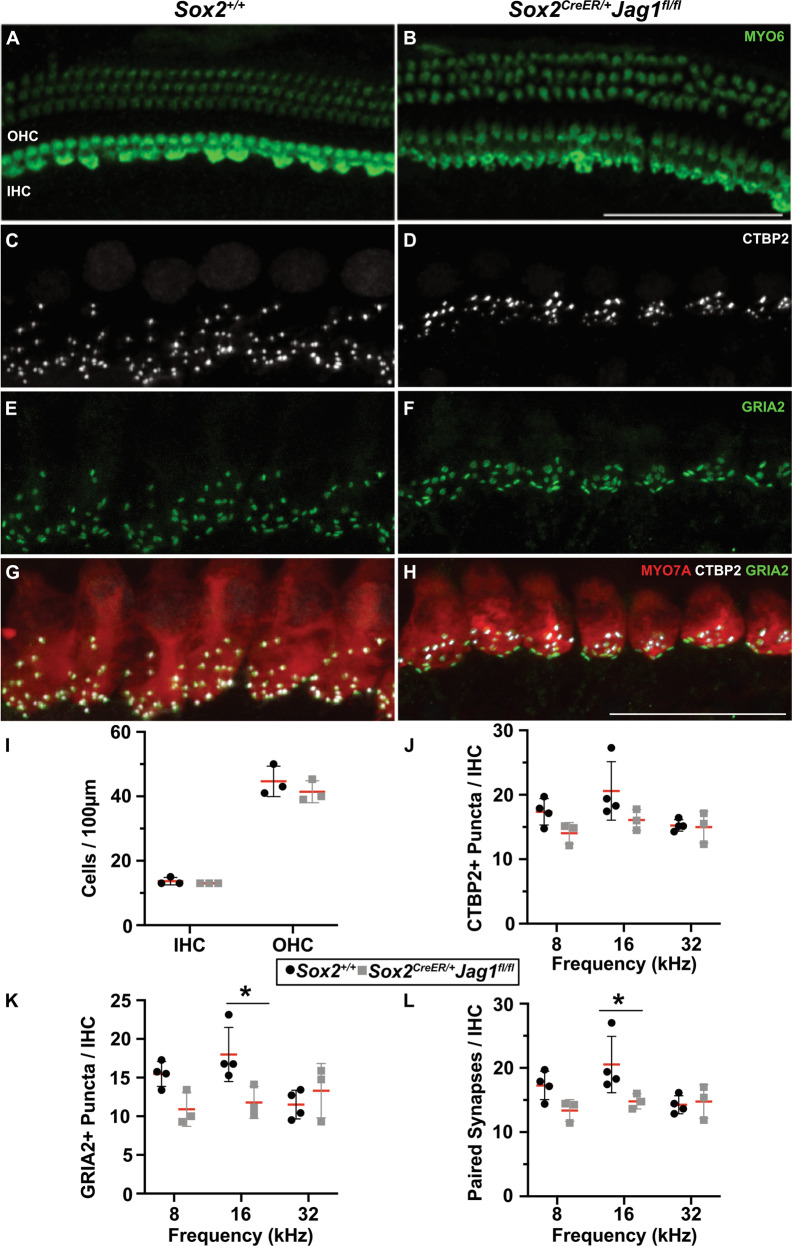
Inner hair cells are present and their synapses are relatively unaffected in JAG1-deleted cochleae at 6 weeks. **A**, **B** Whole mount confocal projections of inner and outer hair cells labeled with Myosin VI (green) at 6 weeks. Scale bar: 100 µm. **C**–**H** Whole mount confocal projections of inner hair cells at 6 weeks. White: CTBP2 presynaptic ribbons; green: GRIA2 postsynaptic receptor patches; red: MYO7A inner hair cells. Scale bar: 25 µm. **I** Average quantities of inner (IHC) and outer hair cells (OHC) between *Sox2*^*+/+*^ littermate controls (black) and *Sox2*^*CreER/+*^*Jag1*^*fl/fl*^ mutants (gray) are not significantly different at 6 weeks of age. Two-way ANOVA Bonferroni adjusted; Data expressed as mean (red bars) ± SD; *n* = 3 per genotype. **J**–**L** Quantification of synaptic components per inner hair cell at 8, 16, and 32 kHz. Data expressed as mean (red bars) ± SD; significance (*) indicated *Sox2*^*+/+*^ vs. *Sox2*^*CreER/+*^*Jag1*^*fl/fl*^; two-way ANOVA Bonferroni adjusted. *Sox2*^*+/+*^ *n* = 4, *Sox2*^*CreER/+*^*Jag1*^*fl/fl*^ *n* = 3. **J** No significant difference was observed in the average amount of CTBP2 + presynaptic puncta per inner hair cell of *Sox2*^*CreER/+*^*Jag1*^*fl/fl*^ mice (gray squares) compared to *Sox2*^*+/+*^ littermate controls (black circles). **K** A mild reduction in the average amount of GRIA2 + postsynaptic puncta per inner hair cell (*P = 0.02) and **L** paired ribbon synapses (*P = 0.03) were observed at 16 kHz in *Sox2*^*CreER/+*^*Jag1*^*fl/fl*^ mice (gray squares) compared to *Sox2*^*+/+*^ littermate controls (black circles).

Auditory synaptopathies are another class of sensorineural hearing loss and are caused by loss of inner hair cells or their synapses (reviewed in [31]). To investigate if the elevated ABR thresholds (Fig. 2A, red) and decreased wave I amplitudes (Fig. S2A, B, red) of *Sox2*^*CreER/+*^*Jag1*^*fl/fl*^ mice were caused by a reduction in inner hair cells or their synapses, we stained whole mount cochleae for markers of hair cells ((MYO6) (Fig. 4A, B, green) or (MYO7A) (Fig. 4G, H, red)), presynaptic ribbons (CTBP2) (Fig. 4C, D, white) and postsynaptic receptor patches (GRIA2) (Fig. 4E, F, green) at 6 weeks of age. *Sox2*^*CreER/+*^*Jag1*^*fl/fl*^ mice (Fig. 4I, gray squares) had quantities of inner and outer hair cell that were comparable to *Sox2*^*+/+*^ littermate controls (Fig. 4I, black circles), indicating that hair cell loss is not a consequence of JAG1-deletion and is therefore not the cause of increased auditory thresholds. Furthermore, quantification of *Sox2*^*CreER/+*^*Jag1*^*fl/fl*^ auditory synapses revealed only mild reductions in paired synapses at one frequency (16 kHz) compared to *Sox2*^*+/+*^ littermate controls (Fig. 4G, H, L). Given that it has been reported that mice can lose up to 50% of their synapses without major effects on hearing thresholds [32], these mild synaptic losses cannot account for the significant threshold shifts observed in *Sox2*^*CreER/+*^*Jag1*^*fl/fl*^ mice.

### Supporting cells show some alterations in *Sox2*^*CreER/+*^*Jag1*^*fl/fl*^ mice

**Fig. 5:**
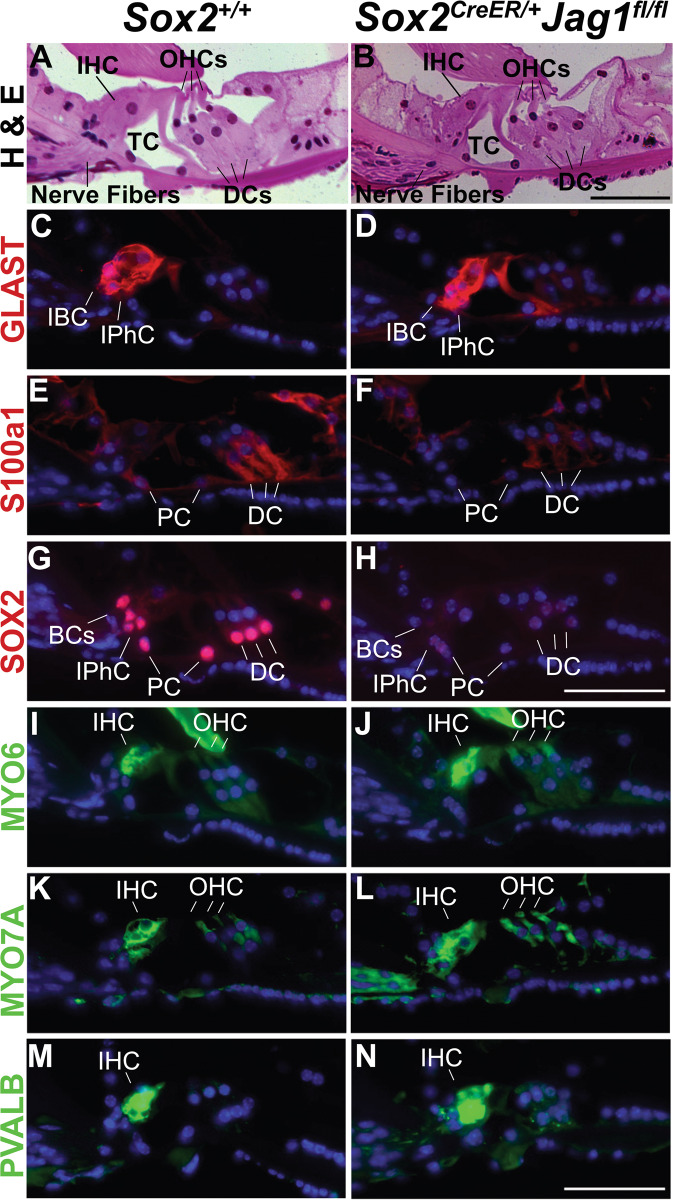
The overall structure of the organ of Corti appeared normal at 6 weeks, although some reduction in supporting cell markers was detected. **A**, **B** 6-week-old plastic sections stained with hematoxylin and eosin show gross cochlear morphology in *Sox2*^*CreER/+*^*Jag1*^*fl/fl*^ mice (**B**) that is similar to *Sox2*^*+/+*^ littermate controls (**A**). Abbreviations: IHC, inner hair cells; OHCs, outer hair cells; DCs, Deiters’ cells; TC, tunnel of Corti. Scale bar: 100 µm. **C**–**H** Paraffin sections of 6-week-old cochlea stained for supporting cells (red) and nuclei (DAPI). Scale bar: 50 µm. **C** *Sox2*^*+/+*^ littermate control sections and (**D**) *Sox2*^*CreER/+*^*Jag1*^*fl/fl*^ mutants showing the normal expression of GLAST in IBC and IPhC. **E** *Sox2*^*+/+*^ littermate control sections showing the normal expression of S100a1 in PC and DC. **F** *Sox2*^*CreER/+*^*Jag1*^*fl/fl*^ mutant sections showing decreased expression of S100a1 in PC and DC. **G** *Sox2*^*+/+*^ littermate control sections showing the normal expression of SOX2 in all supporting cell subtypes. **H** *Sox2*^*CreER/+*^*Jag1*^*fl/fl*^ mutant sections showing decreased expression of SOX2 in all supporting cell types. **I**–**N** Paraffin sections through 6-week-old cochlea stained for hair cells (green), and nuclei (DAPI). Scale bar: 50 µm. **I** *Sox2*^*+/+*^ littermate control sections and (**J**) *Sox2*^*CreER/+*^*Jag1*^*fl/fl*^ mutants showing the normal expression of MYO6 in IHC and OHC. (K) *Sox2*^*+/+*^ littermate control sections and (**L**) *Sox2*^*CreER/+*^*Jag1*^*fl/fl*^ mutants showing the normal expression of MYO7A in IHC and OHC. **M** *Sox2*^*+/+*^ littermate control sections and (**N**) *Sox2*^*CreER/+*^*Jag1*^*fl/fl*^ mutants showing the normal expression PVALB in IHCs. IHC inner hair cells, OHCs outer hair cells, IPhC inner phalangeal cells, IPC inner pillar cells, OPC outer pillar cells, BCs border cells, IBC inner border cells, DCs Deiters’ cells.

Since JAG1 is localized to cochlear supporting cells, we wanted to examine the effects of *Jag1* deletion on supporting cell development. Structurally, the organ of Corti appeared largely normal, complete with the tunnel of Corti, formed by the supporting pillar cells (Fig. 5B). Previously, a group reported that deletion of JAG1 results in substantial loss of Hensen’s cells (HeC), a supporting cell subtype located distally to the outer hair cells, without causing significant effects on cochlear function [33]. Thus, we wanted to determine if the hearing loss in our JAG1 mutants was a consequence of alterations in other supporting cell subtypes, particularly those located adjacent to the inner hair cells. To this end, we performed immunohistochemistry on 6-week-old sections stained with supporting cell-specific markers (GLAST, S100a1, SOX2) (Fig. 5C–H, red) and hair cell-specific markers (MYO6, MYO7A, PVALB) (Fig. 5I–N, green). Results showed that supporting cells markers were present, although in some cases (S100a1 and SOX2) exhibited reduced expression in *Sox2*^*CreER/+*^*Jag1*^*fl/fl*^ cochleae (Fig. 5F, H, red). Additionally, *Sox2*^*CreER/+*^*Jag1*^*fl/fl*^ mice maintained expression of all three hair cell markers analyzed (Fig. 5J, l, N, green). These results indicated that hair cells and the supporting cells immediately surrounding them are present in *Sox2*^*CreER/+*^*Jag1*^*fl/fl*^ mice. To determine if we saw similar changes in the distal Hensen’s cell population as previously reported [33], we performed immunohistochemistry at P6 using markers of hair cells and supporting cells (Fig. S3). Quantification revealed *Sox2*^*CreER/+*^*Jag1*^*fl/fl*^ mice had substantial Hensen’s cell loss in all cochlear turns (Fig. S3I, HeC, red squares). Taken together, these results suggest that JAG1 deletion in the maturing cochlea may affect some aspects of supporting cell development and/or maintenance. However, despite the reduction in some supporting cell marker expression and in Hensen’s cells, these alterations have not been reported to cause significant hearing loss [33]. Moreover, the lack of effects on outer hair cell function (measured by DPOAEs) in *Sox2*^*CreER/+*^*Jag1*^*fl/fl*^ mice despite the abnormalities in the outer supporting cell region also supports the idea that these alterations are unlikely to underlie the increased threshold shifts detected by ABR in *Sox2*^*CreER/+*^*Jag1*^*fl/fl*^ mice.

### The Rho GTPase signaling pathway is dysregulated in JAG1-deleted cochleae

**Fig. 6:**
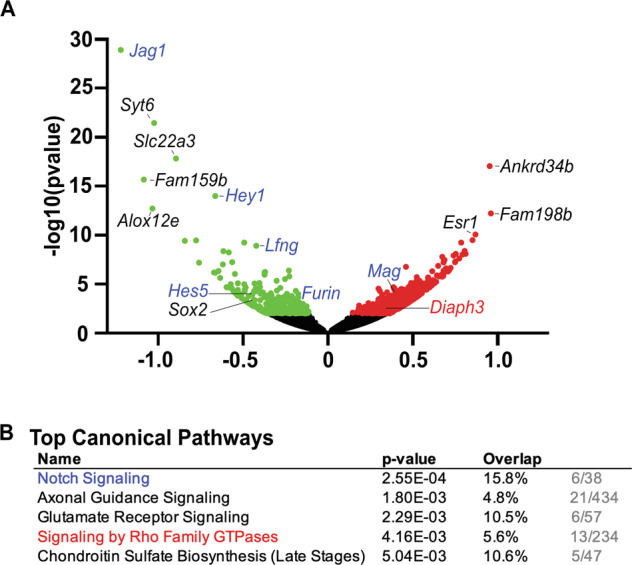
Transcriptional analyses indicate a potential defect in stereocilia formation. **A** Volcano plot from RNA-seq analysis of P6 *Sox2*^*CreER/+*^*Jag1*^*fl/fl*^ and *Sox2*^*+/+*^ littermate cochleae (*n* = 6 per genotype). A total of 411 genes were significantly upregulated (red dots) and 137 genes were significantly downregulated (green dots). Genes in the Notch pathway that show significant differences are indicated in blue. *Diaph3*, a gene in the Rho GTPase family, is shown in red. **B** Top canonical pathways affected by loss of JAG1 identified by Ingenuity Pathway Analysis (IPA). Five of the top pathways whose gene members were significantly different between *Sox2*^*Cre/+*^*Jag1*^*fl/fl*^ and *Sox2*^*+/+*^. Overlap indicated the percentage of genes affected in each pathway (actual numbers are shown in gray).

To provide additional insights into the role that JAG1 plays during cochlear maturation and to identify potential molecular differences, we performed RNA-seq on the cochlear sensory epithelium of P6 *Sox2*^*CreER/+*^*Jag1*^*fl/fl*^ mutant and *Sox2*^*+/+*^ littermate control mice after tamoxifen administration at P0/P1. We found 548 differentially expressed genes with an adjusted p-value of <0.05, 411 of those genes were significantly upregulated (Fig. 6A, red) while 137 were significantly downregulated (Fig. 6A, green; datasets D1 and D2). Ingenuity Pathway Analysis (IPA; Qiagen) revealed a number of different pathways that were significantly altered by loss of JAG1 in the cochlea. Not surprisingly, Notch Signaling was the top pathway affected by loss of JAG1 (Fig. 6A, B, blue). However, one of the top four pathways, signaling by Rho family GTPases, was of interest to us as this family is known to regulate the actin cytoskeleton [34, 35], an important component of hair cell structure, particularly the stereocilia. Notably, one of the genes in this pathway that was significantly differentially overexpressed was *Diaph3*, a member of the diaphanous-related formin (DRF) family involved in actin remodeling (reviewed in [36]). Importantly, pathogenic variants in the *Diaph3* gene in humans leads to DIAPH3 overexpression and causes autosomal dominant auditory neuropathy (AUNA1) [37], a specific form of hearing loss that resembles the type we observed in the *Sox2*^*CreER/+*^*Jag1*^*fl/fl*^ mutants (Fig. 2). Moreover, mouse models of AUNA1 that have been generated by overexpressing *Diaph3* show stereocilia fusion specifically affecting the inner hair cells [38, 39]. Taken together, these similarities to AUNA1 indicated that potentially, actin dysregulation and stereocilia defects were underlying the hearing loss in the *Sox2*^*CreER/+*^*Jag1*^*fl/fl*^ mutant cochleae.

### *Sox2*^*CreER/+*^*Jag1*^*fl/fl*^ inner hair cells display dysmorphic stereocilia

**Fig. 7:**
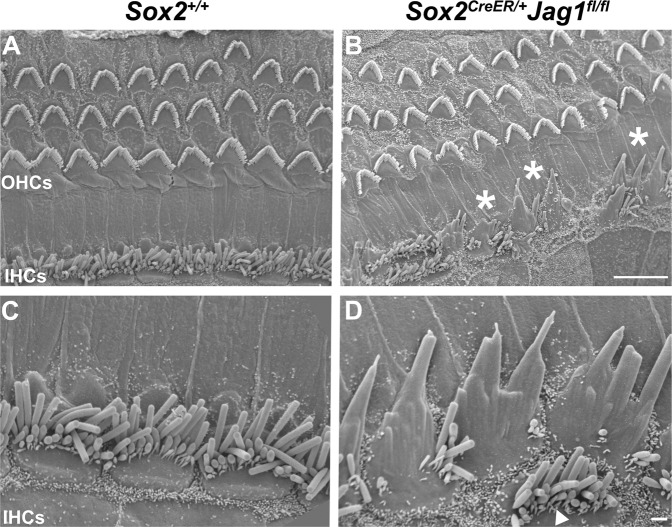
*Sox2*^*CreER/+*^*Jag1*^*fl/fl*^ inner hair cells display stereocilia malformations. **A**–**D** Scanning electron microscopy (SEM) of the sensory regions of 6-week-old *Sox2*^*CreER/+*^*Jag1*^*fl/fl*^ and *Sox2*^*+/+*^ littermate cochleae. The majority of *Sox2*^*CreER/+*^*Jag1*^*fl/fl*^ inner hair cells (**B**; asterisks) display significant stereocilia fusion compared to *Sox2*^*+/+*^ littermate controls (**A**), although there are some hair cells with unfused stereocilia (**D**; arrowhead). However, outer hair cell stereocilia of the mutant mice appear to have normal morphology. Scale bar: 10 µm. **C**, **D** Higher power images of *Sox2*^*CreER/+*^*Jag1*^*fl/fl*^ inner hair cells further detail stereocilia fusion compared to *Sox2*^*+/+*^ littermate controls. Scale bar: 1 µm.

To examine whether JAG1-deficiency caused stereocilia defects in hair cells, we used scanning electron microscopy (SEM) at 6 weeks of age to examine the ultrastructure of hair cell stereocilia. Dramatic malformations of the inner hair cell stereocilia were observed in *Sox2*^*CreER/+*^*Jag1*^*fl/fl*^ cochleae (Fig. 7B) compared to *Sox2*^*+/+*^ littermate controls (Fig. 7A). Specifically, we found that *Sox2*^*CreER/+*^*Jag1*^*fl/fl*^ inner hair cell stereocilia often appeared fused, such that individual stereocilia were not recognizable (Fig. 7B, asterisks). This stereocilia fusion was observed in ~70% of *Sox2*^*CreER/+*^*Jag1*^*fl/fl*^ inner hair cells throughout the cochlea (Fig. S4). However, these defects were mostly prevalent in the apical/middle cochlear turns (Fig. S4A–I) and less frequently observed in the more basal regions (Fig. S4J, K). In contrast, outer hair cell stereocilia appeared to have normal morphology throughout the cochlea (Fig. 7B), indicating that these defects were specific to inner hair cells. Taken together, these results were consistent with results of the auditory testing (Fig. 2), which indicated that the inner hair cell pathway was disrupted in JAG1-deficient mice, and are consistent with AUNA1 mouse models that displayed fused stereocilia bundles, caused by overexpression of *Diaph3*.

## Discussion

During embryonic inner ear development, the Notch ligand JAG1 plays an established role in prosensory region specification through the process of lateral induction [2, 7–10]. Although several of the Notch ligands are expressed in the cochlea, including DLL1 and JAG2 [11, 14, 40], JAG1 is the only ligand expressed in supporting cells and maintained in the mature cochlea [16]. However, the function of JAG1 in the maturing cochlea is not well characterized. Here, we show that deletion of JAG1 in the cochlea results in hearing loss that resembles auditory neuropathy. Specifically, we show that ABR thresholds are significantly increased while DPOAEs, which assess outer hair cell function, are normal in *Sox2*^*CreER/+*^*Jag1*^*fl/fl*^ mice. These results indicate that the inner hair cell pathway is primarily affected in *Sox2*^*CreER/+*^*Jag1*^*fl/fl*^ mice. Analysis of the cellular makeup of the organ of Corti shows that hair cells and supporting cells are largely present, as well as auditory neurons in *Sox2*^*CreER/+*^*Jag1*^*fl/fl*^ mice. Moreover, inner hair cell synapses are also largely unaffected in *Sox2*^*CreER/+*^*Jag1*^*fl/fl*^ mice. Molecular analysis at P6 using RNA-seq indicated that the Rho GTPase signaling pathway, including *Diaph3*, is significantly altered in JAG1-deficient cochlea. Previous studies have shown that *Diaph3* overexpression causes auditory neuropathy in humans (AUNA1), and mouse models revealed inner hair cell stereocilia defects. When analyzed by SEM, JAG1-deficient cochleae display similar defects in the inner hair cell stereocilia. Taken together, our data show that deletion of JAG1 during cochlear maturation results in hearing loss caused by inner hair cell stereocilia defects, resembling AUNA1 mouse models.

In the cochlea, Notch signaling plays a well-established role in mediating lateral inhibition, which determines whether a cochlear precursor will adopt a hair cell or supporting cell fate. Disruptions in lateral inhibition during embryogenesis result in an overproduction of hair cells at the expense of supporting cells [11–13]. Additionally, disruptions in Notch signaling during the early postnatal period can also result in excess hair cells [41–44]. However, we did not observe excess hair cell production after JAG1 deletion, indicating that JAG1 signaling does not participate in lateral inhibition during cochlear maturation. Another role that Notch can play is an instructive role during differentiation. This role is less well understood but has been demonstrated in the central nervous system, where Notch has been shown to promote the differentiation of glia [45–51]. Given that supporting cells share some similarities with glia (reviewed in [52]), it is possible that JAG1 plays an instructive role in supporting cell differentiation. In support of an instructive role, we have previously shown that ectopically activating Notch in early-developing hair cells can convert them to a supporting cell-like fate [19]. Similarly, other groups have shown that overactivation of Notch promotes supporting cell-specific gene expression and Deiters’ cell loss [22]. Most recently, Chrysostomou et al., (2020) showed that deleting JAG1 in the early developing cochlea (*Sox2*^*CreERT2/+*^:: *Jag1*^*fx/fx*^ *or Fgfr3-iCreER*^*T2*^:: *Jag1*^*fx/fx*^) results in loss of Hensen’s cells, a distal supporting cell population that flanks the third row of outer hair cells [33]. We also observed a loss of Hensen’s cells after JAG1 deletion (*Sox2*^*CreER/+*^*Jag1*^*fl/fl*^) at P0/P1. However, Chrysostomou showed that despite lacking most Hensen’s cells, there was little effect on hearing thresholds using a Cre allele that only deleted JAG1 in the outer supporting cells (*Fgfr3-iCreER*^*T2*^:: *Jag1*^*fx/fx*^) [33]. Thus, these results indicate that the presence of Hensen’s cells is not critical for maintaining hearing thresholds. We show here that JAG1 deletion throughout the supporting cell population (*Sox2*^*CreER/+*^*Jag1*^*fl/fl*^) results in significant increases in ABR thresholds. Even more, interestingly, there were no effects on *Sox2*^*CreER/+*^*Jag1*^*fl/fl*^ DPOAEs, an assessment of outer hair cell function. These results indicate that JAG1 plays another role in the cochlea outside of regulating Hensen’s cell numbers, which specifically affects inner hair cell function.

Clinically, patients with hearing loss that present with increased ABR thresholds and normal DPOAEs would be classified as having auditory neuropathy, indicating inner hair cell function is compromised, while outer hair cell function is maintained. In mice, hearing loss is commonly classified as either a synaptopathy (caused by loss of inner hair cell synapses), a neuropathy (caused by loss of spiral ganglion neurons/ dysfunction of the auditory nerve), an amplifier defect (caused by loss of outer hair cells) or a global dysfunction (caused by improper homeostatic /ionic balance) (reviewed in [53]). Our physiological and histological analysis indicate that JAG1-deficient cochleae have normal inner hair cell numbers, relatively normal inner hair cell synapses, and normal numbers of auditory neurons, ruling out these as reasons for the increased auditory thresholds. Instead, our molecular results using RNA-seq analysis indicate that the Rho GTPase signaling pathway is disrupted in *Sox2*^*CreER/+*^*Jag1*^*fl/fl*^ mice, a pathway commonly involved in actin dynamics. Since the stereocilia are composed mainly of actin, and stereocilia defects are one of the most common causes of deafness [54], we used SEM to demonstrate that the inner hair cell stereocilia were abnormal in *Sox2*^*CreER/+*^*Jag1*^*fl/fl*^ mice, often showing fused and elongated stereocilia. The fact that only the inner hair cell stereocilia are abnormal is consistent with physiological results in JAG1-deficient mice showing raised ABR thresholds while maintaining normal DPOAEs, indicating normal outer hair cell function. Importantly, we are deleting JAG1 at P0/P1 which is an early stage of stereocilia development when the stereocilia are not fully organized or mature. Studies have shown that in rodents, postnatal stages are a critical time for stereocilia morphogenesis, including elongation, significant microvilli reabsorption, and staircase refinement [55]. Additionally, the kinocilia of mouse auditory hair cells, an important mediator of hair cell morphogenesis and planar cell polarity (PCP), continually degenerates until its absence at the onset of hearing (postnatal day (P)12) (reviewed in [56]). Previously it has been shown that members of the Rho GTPase family play an essential role in cochlear hair cell stereocilia formation and maintenance. Specifically, loss or dysregulation of the Rho GTPases RAC1 [57], CDC42 [58, 59], their activator ARHGEF6 [60], or their downstream effector PAK1 [61] leads to deficits in cochlear stereocilia development or maintenance. Interestingly, others have shown PCP dysregulation in hair bundles after *Cdc42* deletion in both hair cells and supporting cells, but this was not observed after hair cell-only *Cdc42* deletion [58, 62]. Furthermore, recent results from Du et al. (2021) demonstrate that CDC42 acts in both a cell-autonomous and non-autonomous manner during stereocilia development [59]. Specifically, a more severe stereocilia phenotype with additional planar cell polarity (PCP) defects were observed after treatment with a CDC42 inhibitor compared to the stereocilia phenotype observed after hair cell-specific *Cdc42* inactivation, indicating involvement of other cells like supporting cells, in *Cdc42*-mediated PCP regulation [59]. These studies indicate that cochlear supporting cells likely play a role in hair cell stereocilia formation and maintenance.

We also identified a gene of interest, *Diaph3*, within the signaling by Rho family GTPase pathway that was significantly upregulated in JAG1-deficient cochleae. *Diaph1-3* are members of the mammalian diaphanous-related formin (DRF) family that are activated by Rho GTPases, and are involved in actin remodeling [63–65]. Importantly, pathogenic variants in DIAPH1 and DIAPH3 have been shown to cause deafness. Previously, studies in humans [66–69] and mice [70, 71] have identified that pathogenic variants in DIAPH1 cause DFNA1, a form of non-syndromic autosomal dominant sensorineural hearing loss. These pathogenic variants are thought to interfere with the autoregulation of DIAPH1, resulting in a constitutively active protein [65, 66]. Similarly, work conducted in both humans and mice has also established a link between *Diaph3* levels and hearing loss. In humans, heterozygous mutation in the *DIAPH3* gene cause autosomal dominant auditory neuropathy 1 (AUNA1). Specifically, a point mutation in the 5’ untranslated region of the *DIAPH3* human gene leads to overexpression of the DIAPH3 protein [37]. Mouse models of AUNA1, in which *Diaph3* is overexpressed, exhibit auditory neuropathy hearing loss [38, 39]. Strikingly, mouse models of *Diaph3* overexpression show abnormal inner hair cell stereocilia morphology [38], similar to the morphology of JAG1-deficient inner hair cells. Taken together, these studies indicate that overexpression of *Diaph3* is deleterious for hearing.

Currently, the identity and cellular location of the Notch receptor(s) mediating the effects of JAG1 in the maturing cochlea are unknown. However, out of the four Notch receptors (NOTCH1-4), three have reported expression in the organ of Corti (*Notch1-3*) [15, 40]. Previous studies have shown that NOTCH1 plays a role in mediating lateral inhibition postnatally, and in preventing continued proliferation [72, 73]. NOTCH2 is also a possible receptor given that it acts as the receptor for JAG1 in other systems that are affected in Alagille syndrome [74]. Thus, NOTCH1 and NOTCH2 are possible receptors based on established roles in the cochlea or other systems, but NOTCH1, NOTCH2, NOTCH3, or a combination of these receptors, could mediate the effects of JAG1 in the maturing cochlea. Our results uncover a novel role for JAG1 in the maturing cochlea in maintaining the inner hair cell stereocilia. Given that JAG1 is expressed in supporting cells and likely receptors are also in supporting cells [15, 40, 75], these results highlight a potential interaction between hair cell and supporting cells that is required to maintain the stereocilia. Future studies will reveal how the Notch pathway interacts with the Rho GTPase signaling pathway to regulate stereocilia maintenance.

## Supplementary information

Supplemental File Information

Supplemental Figure 1

Supplemental Figure 2

Supplemental Figure 3

Supplemental Figure 4

Supplemental Table 1

checklist

Dataset 1

Dataset 2

## Data Availability

The dataset(s) supporting the conclusions of this article is(are) included within the article (and its additional file(s)). The sequencing dataset discussed in this publication is deposited at NCBI’s Gene Expression Omnibus (GEO accession number GSE193158).
