# Supplementary material for: Deletion of the Notch ligand Jagged1 during cochlear maturation leads to inner hair cell defects and hearing loss
Source: Cell Death Dis. 2022 Nov 18;13(11):971. doi: 10.1038/s41419-022-05380-w (PMC9674855; doi:10.1038/s41419-022-05380-w)
Supplement: Supplementary file 1 — Supplemental File Information [file 41419_2022_5380_MOESM1_ESM.docx]

**Supplementary File Information**

**Supplementary Figure 1.** Deletion of the Notch ligand JAG1 in maturing cochlea results in decreased protein expression in supporting cells.

(A) Drawing of a cross-section through the neonatal organ of Corti showing JAG1 expression in supporting cells (red) at postnatal day (P)6. JAG1 is highly expressed in supporting cells, including PC, IPhC and DC (red). JAG1 is not expressed in IHC or OHC (white). Abbreviations: IHC, inner hair cells; OHCs, outer hair cells; IPhC, inner phalangeal cells; IPC, inner pillar cells; OPC, outer pillar cells; DC, Deiters’ cells; TM, tectorial membrane. (B) Experimental strategy. Offspring from *Sox2^CreER/+^Jag^+/fl^* x *Jag1^fl/fl^* crosses were administered tamoxifen (+tmx) at postnatal days (P)0 and P1 and their cochleae were harvested and analyzed at P6. (C-D) Cryosections through P6 cochlea stained for hair cells (MYO6), nuclei (DAPI) and JAG1. Scale bar: 50µm. (C) *Sox2^+/+^* littermate control sections showing the normal expression of JAG1 at P6 in supporting cells. (D) In *Sox2^CreER/+^Jag1^fl/fl^* cochlea at P6 there is significant downregulation of JAG1 protein in all supporting cells.

**Supplementary Figure 2.** Decreased ABR wave I amplitudes are observed in *Sox2^CreER/+^Jag1^fl/fl^* mice at 6 weeks.

(A) Mean ABR waveform traces obtained in response to a 75dB pure tone stimulus at 16kHz for control (black) and *Sox2^CreER/+^Jag1^fl/fl^* mutants (red) mice at 6 weeks. Substantial alterations in the ABR waveform, particularly in wave I, are observed in *Sox2^CreER/+^Jag1^fl/fl^* (red) compared to controls (black). ABR waves are labeled above the traces with Roman numerals I-V. *Sox2^+/+^* n=7, *Sox2^CreER/+^Jag1^fl/fl^* n=6. (B) ABR wave I amplitudes at increasing sound pressure levels (dB SPL) at 16kHz. *Sox2^CreER/+^Jag1^fl/fl^* mutant mice (red) do not display linear increases in wave I responses at increasing sound pressure levels compared to the control mice (black). Data expressed as mean ± SEM. *Sox2^+/+^* n=11, *Sox2^CreER/+^Jag1^fl/fl^* n=12. Two-way ANOVA Bonferroni adjusted; ****P*≤0.001.

**Supplementary Figure 3.** Deletion of *Jag1* in the maturing cochlea causes loss of Hensen’s cells by P6, although other cell types are not affected.

(A-H) Whole mount confocal images of P6 cochlea. Scale bar: 50µm. (A-B) Phalloidin (magenta) labels F-actin within the inner and outer hair cell stereocilia of P6 *Sox2^+/+^* littermate control (A) and *Sox2^CreER/+^Jag1^fl/fl^* (B) mice. (C-H) SOX2 immunostaining (green) labels the nuclei of all supporting cell subtypes, except CCs. Arrowheads denote the location of HeCs. (C-F) CD44 immunostaining labels CCs and OPCs. FABP7 immunostaining labels IPhCs, HeCs and BCs; which is lacking in *Sox2^CreER/+^Jag1^fl/fl^* mutant cochleae in the HeC region (F; arrowhead). (G-H) PROX1 labels OPCs and three rows of DCs. Dotted line designates the boundary between HeCs and CCs. (I) Quantification of hair cell and supporting cell subtypes. Quantities of inner and outer hair cells are similar between *Sox2^CreER/+^Jag1^fl/fl^* (red squares) and controls (black circles). Substantial HeC loss is observed in *Sox2^CreER/+^Jag1^fl/fl^* mutant cochleae. Data expressed as mean ± SD; Two-way ANOVA Bonferroni adjusted; *** *P* ≤0.001; n=3 per genotype. Abbreviations: HC, hair cell; SC, supporting cell; IHC, inner hair cell; OHCs, outer hair cell; IPhC, inner phalangeal cell; IPC, inner pillar cell; OPC, outer pillar cell; DCs, Deiters’ cells; HeCs, Hensen’s cells; CCs, Claudius cells.

**Supplementary Figure 4.** Additional examples of inner hair cell stereocilia malformations along the length of *Sox2^CreER/+^Jag1^fl/fl^* cochleae.

Representative scanning electron microscopy (SEM) of the sensory regions of 6-week-old *Sox2^CreER/+^Jag1^fl/fl^* cochleae from apex (A) to the base (L). (A,D,G,J) Lower power images of inner and outer hair cells highlight morphological differences observed throughout each cochlear turn. Scale Bars: 10µm. The majority of *Sox2^CreER/+^Jag1^fl/fl^* inner hair cells (IHCs) display significant stereocilia malformations such as fusions and elongations (asterisks), although there are some hair cells with unfused stereocilia that are more prevalent in the basal turns (J-L). (B,C,E,F,H,I,K,L) Higher magnification images of *Sox2^CreER/+^Jag1^fl/fl^* inner hair cells (IHCs) further detail the stereocilia malformations (asterisks) that are present throughout the cochlea. Scale Bars: 5µm.

**Supplementary Table 1.** Table of antibodies used in this study.
