# Supplementary figures and images for: Deletion of the Notch ligand Jagged1 during cochlear maturation leads to inner hair cell defects and hearing loss
Source: Cell Death Dis. 2022 Nov 18;13(11):971. doi: 10.1038/s41419-022-05380-w (PMC9674855; doi:10.1038/s41419-022-05380-w)

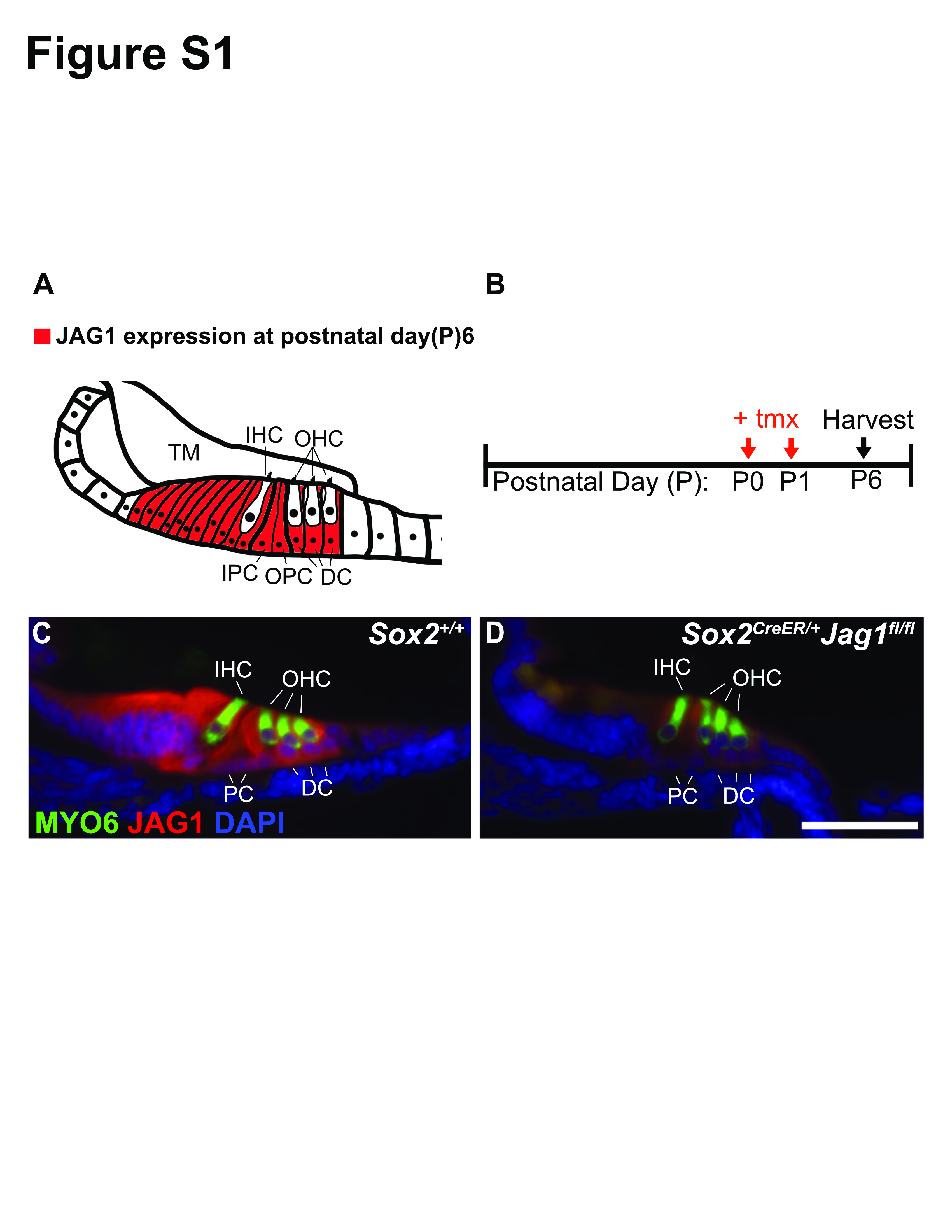

Supplement: Supplementary file 2 — Supplemental Figure 1 [file 41419_2022_5380_MOESM2_ESM.tif]

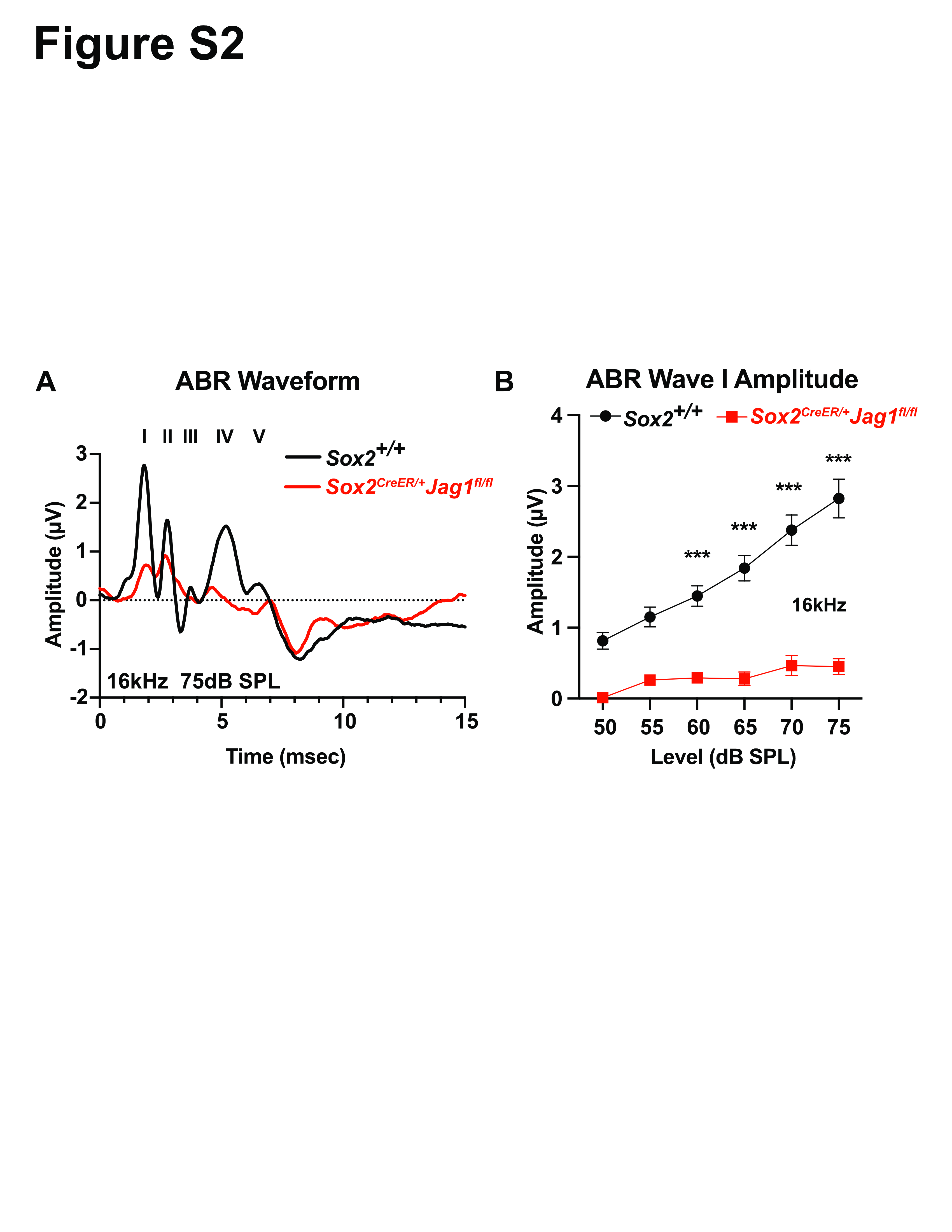

Supplement: Supplementary file 3 — Supplemental Figure 2 [file 41419_2022_5380_MOESM3_ESM.tif]

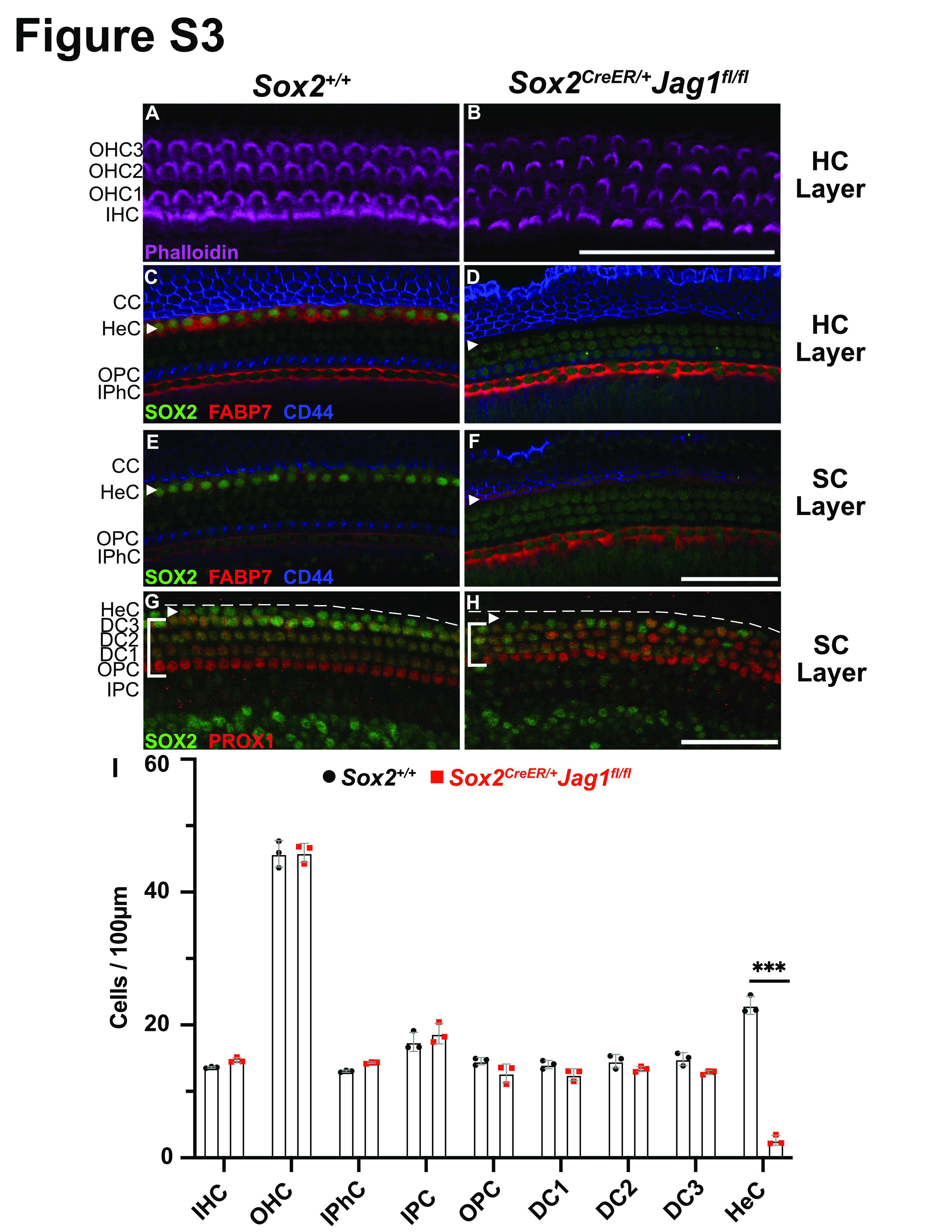

Supplement: Supplementary file 4 — Supplemental Figure 3 [file 41419_2022_5380_MOESM4_ESM.tif]

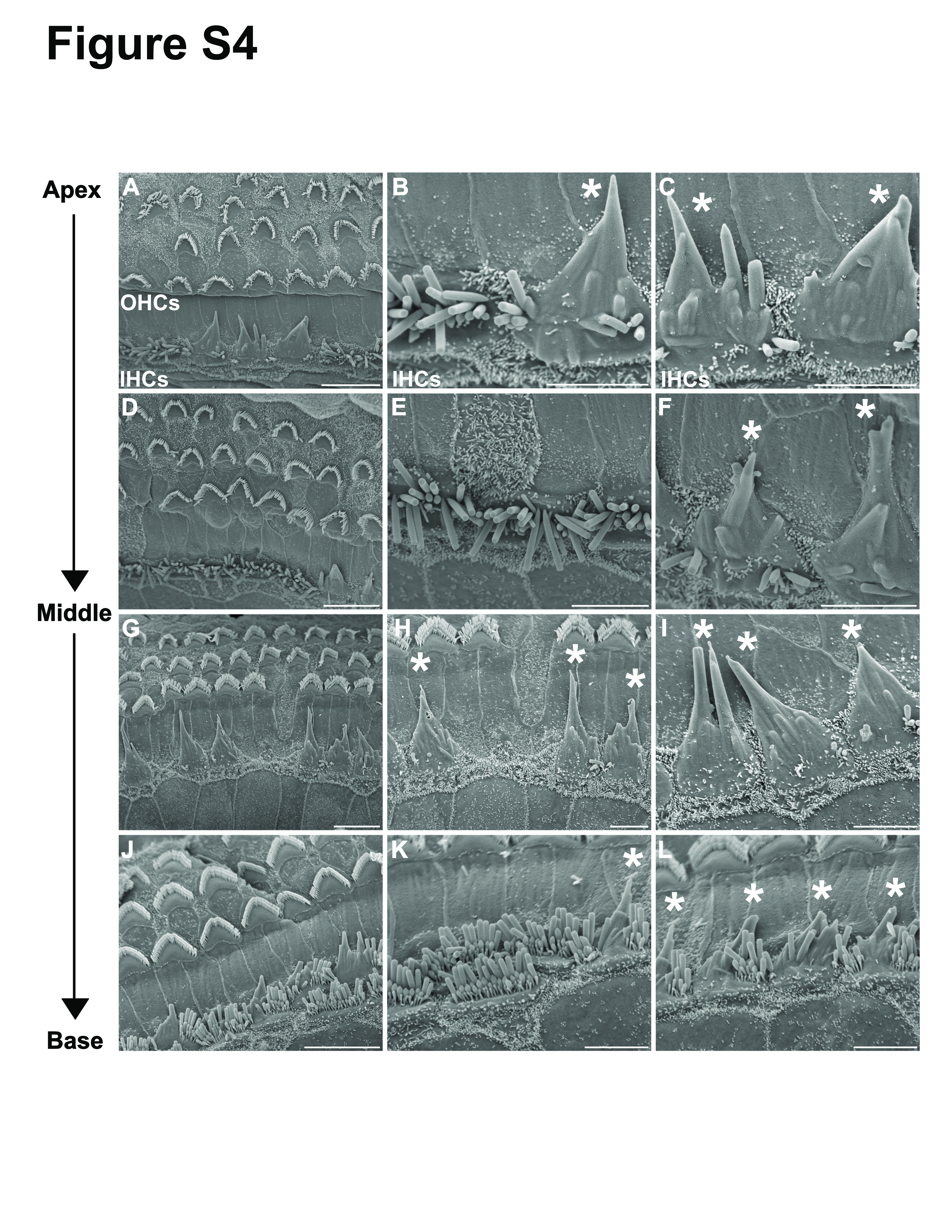

Supplement: Supplementary file 5 — Supplemental Figure 4 [file 41419_2022_5380_MOESM5_ESM.tif]
