# Supplementary material for: Deletion of the Notch ligand Jagged1 during cochlear maturation leads to inner hair cell defects and hearing loss
Source: Cell Death Dis. 2022 Nov 18;13(11):971. doi: 10.1038/s41419-022-05380-w (PMC9674855; doi:10.1038/s41419-022-05380-w)
Supplement: Supplementary file 6 — Supplemental Table 1 [file 41419_2022_5380_MOESM6_ESM.docx]

**Table S1. Primary Antibodies**

| **Antibody** | **Company** | **Cat#** | **RRID** | **Concentration**  **& Incubation** |
| --- | --- | --- | --- | --- |
| CD44 | BD Biosciences | 550538 | AB_393732 | [1:500]  Overnight at 4ºC |
| CtBP2 | BD Transduction Laboratories | 612044 | AB_399431 | [1:200]  Overnight at 37ºC |
| FABP7 | Abcam | ab32423 | AB_880078 | [1:200]  Overnight at 4ºC |
| GLAST (EAAT1) | Abcam | ab416 | AB_304334 | [1:100]  Overnight at 4ºC |
| GluR2 / Gria2 | Millipore | MAB397 | AB_2113875 | [1:2000]  Overnight at 37ºC |
| JAG1 c-20 | Santa Cruz Biotechnology | sc-6011 | AB_649689 | [1:200]  Overnight at 4ºC |
| JAG1 | R&D Systems | AF599 | AB_2128257 | [1:250]  Overnight at 4ºC |
| MYOSIN-VI | Proteus | 25-6791 | AB_10013626 | [1:700]  Overnight at 4ºC |
| MYOSIN-VIIa | Proteus | 25-6790 | AB_10015251 | [1:700]  Overnight at 4ºC |
| PROX1 | Millipore | ABN278 | AB_2811075 | [1:2000]  Overnight at 4ºC |
| PVALB | Sigma | P3088 | AB_477329 | [1:1000]  Overnight at 4ºC |
| S100a1 | Dako | Z0311 | AB_10013383 | [1:50]  Overnight at 4ºC |
| SOX2 | Santa Cruz Biotechnology | sc-17320 | AB_2286684 | [1:700]  Overnight at 4ºC |
| TUJ1 | Covance | mms-435P | AB_2313773 | [1:1000]  Overnight at 4ºC |
